# Green hematite depression for reverse selective flotation separation from quartz by locust bean gum

**DOI:** 10.1038/s41598-023-36104-5

**Published:** 2023-06-02

**Authors:** Mehrdad Kordloo, Gholamreza Khodadadmahmoudi, Ehsan Ebrahimi, Ali Rezaei, Arash Tohry, Saeed Chehreh Chelgani

**Affiliations:** 1 School of Mining Engineering, College of Engineering, University of Tehran, Tehran, Iran; 2 Mining and Metallurgical Engineering Department, Yazd University, Yazd, 89195-741 Iran; 3 Minerals and Metallurgical Engineering, Swedish School of Mines, Department of Civil, Environmental and Natural Resources Engineering, Luleå University of Technology, 971 87 Luleå, Sweden

**Keywords:** Chemical engineering, Environmental impact

## Abstract

Reverse cationic flotation is currently the main processing technique for upgrading fine hematite from silicates. Flotation is known as an efficient method of mineral enrichment that deals with possibly hazardous chemicals. Thus, using eco-friendly flotation reagents for such a process is an emerging need for sustainable development and green transition. As an innovative approach, this investigation explored the potential of locust bean gum (LBG) as a biodegradable depressant for the selective separation of fine hematite from quartz through reverse cationic flotation. Various flotation conditions (micro and batch flotation) were conducted, and the mechanisms of LBG adsorption have been examined by different analyses (contact angle measurement, surface adsorption, zeta potential measurements, and FT-IR analysis). The micro flotation outcome indicated that the LBG could selectively depress hematite particles with negligible effect on quartz floatability. Flotation of mixed minerals (hematite and quartz mixture in various ratios) indicated that LGB could enhance separation efficiency (hematite recovery > 88%). Outcomes of the surface wettability indicated that even in the presence of the collector (dodecylamine), LBG decreased the hematite work of adhesion and had a slight effect on quartz. The LBG adsorbed selectively by hydrogen bonding on the surface of hematite based on various surface analyses.

## Introduction

Due to the substantial iron and steel demands in various industries, low-grade finely disseminated iron oxide ores with complex mineralogy have been accounted as iron resources and processed with different upgrading techniques^1^. It was well-documented that reverse (cationic/anionic) flotation separation is the most comprehensive processing practice for enriching low-grade hematite ores, in which mineral liberations happen in fine fractions^2,3^. Silicates, the most typical gangue phases, would float by cationic/anionic collectors, and hematite should be depressed by depressants^4–6^. However, a massive volume of materials must be fed to the flotation circuits for upgrading hematite from these low-grade ores, which require a substantial quantity of reagents. These facts emerge from using selective and eco-friendly flotation chemicals throughout the process, enhancing process efficiency and reducing potential environmental issues^7,8^. Thus, several investigations have been conducted to explore a green approach for upgrading low-grade hematite ores by considering eco-friendly biodegradable depressants^1,9–17^.

Different depressants such as Starch^18–21^, Dextrin^9,10,17^, Carboxyl Methyl Cellulose^12,13^, humic acids^14,15^, and Tannin^16^ have been successfully examined for such a purpose. These studies indicated that developing environmentally friendly depressants for the reverse flotation separation of hematite would facilitate the green transition toward sustainable development and cleaner production. Thus, it is essential to examine various biodegradable depressants, such as polysaccharide-base, polyphenolic-based, and lignosulfonate-based, for hematite depression and explore their adsorption mechanisms through selective separation.

Locust bean gum (LBG) is a hydrocolloid extracted from the Ceratonia siliqua tree, also known as carob, and is widely used in the food industry^22^. LBG is a galactomannan polysaccharide with high molecular weight and has similar monomeric structures to the guar gum and tara gum^23^. LBG has been successfully used as a depressant for the flotation separation of chalcopyrite from various minerals (Table 1). It was reported that LBG could be selectively depressed by sulfide minerals (sphalerite, pyrite, and galena) and talc. LBG deactivates talc surface by physical adsorption, mainly driven by hydrogen bonding. It would stretch the shear plane of the electrical double layer on the surface of talc particles and reduce their electrical charge magnitude^24^. In contrast, it showed chemical adsorption on the sphalerite surface via interaction with the oxidation products^25^. It was also documented that LBG showed physisorption on the pyrite and galena surface, while this adsorption was weaker on the chalcopyrite particles^23,26^. Surprisingly, the application of LBG as a selective depressant for flotation separation of hematite-quartz has not been reported.

**Table1:** The application of LBG for the depression of minerals.

Depressed mineral	Floated mineral	pH	LBG concentration	Collector name- concentration	Particle size (μm)	Recovery—grade (%)	Ref
Talc	Chalcopyrite	7	2000 g/t	Potassium butyl xanthate (PBX)-(376 g/t)	+ 37–150	Chalcopyrite recovery: 88.1% Cu grade: 30.1% Floated talc < 5%	^24^
Sphalerite	–	7	5 mg/L	Potassium butyl xanthate (PBX)-× 10ˉ^4^ mol/L	+ 37–150	–	^25^
Sphalerite	Chalcopyrite	7	4000 g/t	Potassium butyl xanthate (PBX)-× 10ˉ^4^ mol/L	+ 37–150	Chalcopyrite recovery > 70% floated sphalerite < 5%	^27^
Galena	Chalcopyrite	8	5 mg/L	Sodium butyl xanthate (SBX)-(10 mg/L)	+ 38–74	Chalcopyrite recovery > 80% floated galena < 10%	^26^
Pyrite	Chalcopyrite	8	200 mg/L	Sodium butyl xanthate (SBX)-(10 mg/L)	+ 38–74	Chalcopyrite recovery > 80% floated pyrite < 10%	^23^
Talc	Chalcopyrite	7	200 g/t	Potassium butyl xanthate (PBX)-(80 g/t)	+ 37–150	Cu grade: 20.25% and recovery of 75.8% Floated talc < 5%	^28^

Therefore, as a novel approach, this study is going to examine LBG depression properties for the flotation separation of hematite from quartz during the reverse cationic flotation (by dodecylamine (DDA) as a collector). Single mineral micro-flotation experiments were initially carried out to reveal the effects of LBG on the depression of hematite. Various synthetic hematite and quartz mixtures were used further to explore process selectivity. The wettability of minerals was explored in the absence and presence of LBG and assessed by contact angle measurements at various collector concentrations. Surface characterization was conducted to identify the adsorption mechanism of LBG on both material surfaces.

## Materials and methods

### Minerals

**Figure 1:**
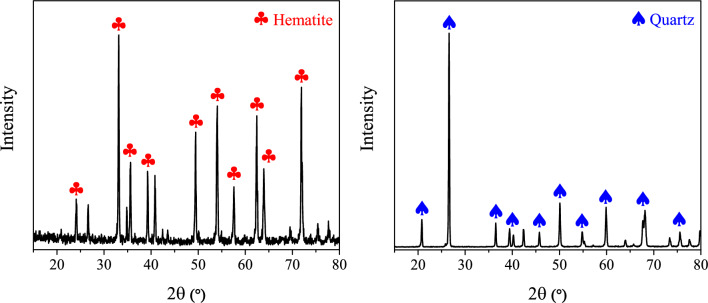
XRD analyses of the hematite and quartz samples.

Hematite and quartz ores were collected from various mining in Kerman and Bandarabas provinces, respectively. A jaw crusher and dry milling were used for crushing bulk samples. The fine particles were sieved, and the particle size distribution of − 75 + 38 μm was applied for micro-flotation. The d_80_ of hematite and quartz were 58 and 62 m$\upmu$, respectively. The hematite sample was not completely pure and enriched with one magnetic separation, followed by a Mozley table to improve the iron grade and remove impurities. The samples were characterized and analyzed by X-ray diffraction (XRD) using a D8 Advance AXS Bruker, and X-ray fluorescence (XRF) using a Perkin Elmer Optima 4300 XRF. The XRD spectra of the hematite and quartz samples (Fig. 1) have confirmed the purity of the minerals. The XRF analysis verified the samples' relatively high purity, the iron content of the hematite sample is 95.5%, and the silica content of the quartz sample is 97.75% (Table 2).

**Table 2:** XRF analyses of the hematite and quartz samples.

Samples	XRF analysis (%)										
	Fe_2_O_3_	SiO_2_	Al _2_O_3_	BaO	K_2_O	MgO	MnO	Na_2_O	P_2_O_5_	TiO_2_	LOI
Hematite Ore	40.13	19.85	2.02	0.01	0.59	3.06	0.21	0.07	0.1	0.4	3.27
Hematite	95.55	2.81	0.48	0.12	0.07	0.08	0.16	0.05	0.05	0.014	0.54
Quartz	1.00	97.75	0.75	–	0.24	–	–	–	–	0.07	0.17

### Reagents

**Figure 2:**
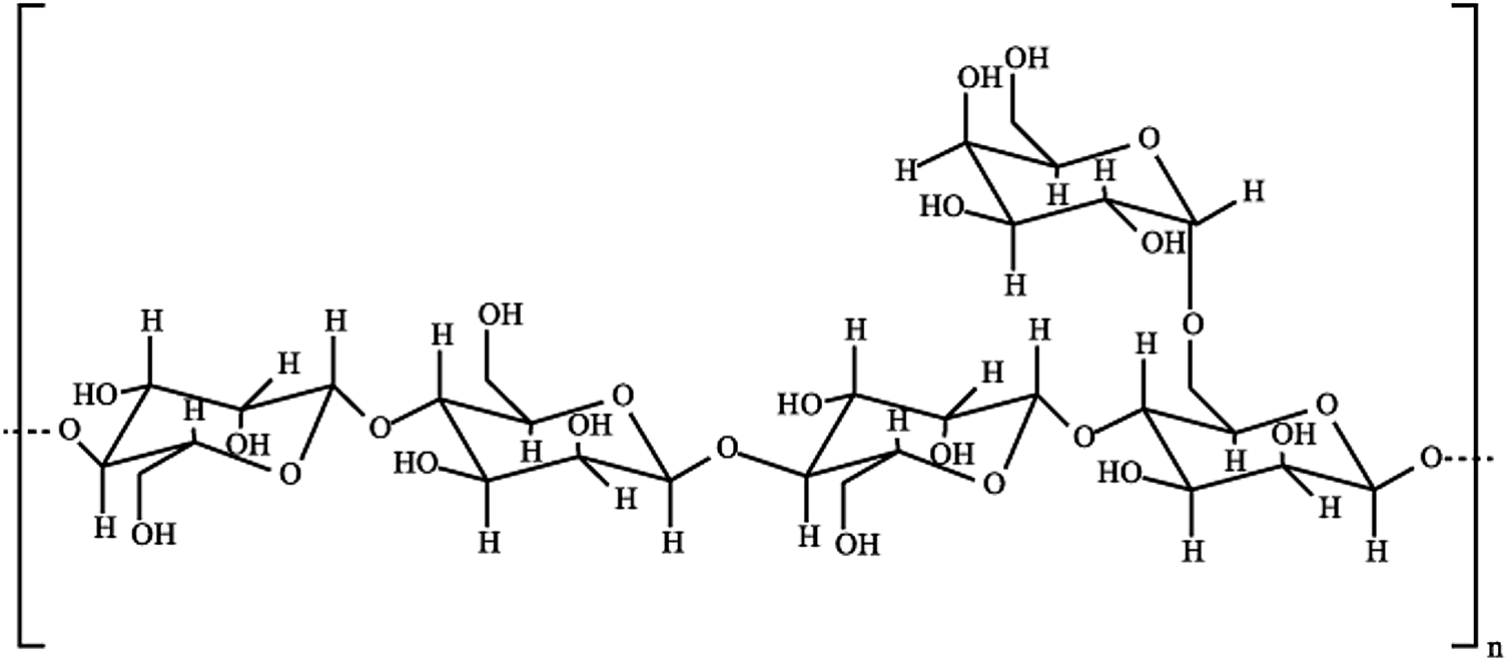
Structural formula of locust bean gum^22^.

LBG (molecular weight 226.66 g/mol, Aldrich grade, < 20 mm) as the hematite depressant was purchased from Pishgaman Company in Tehran, Iran. LBG was available as a solid white powder at room temperature (Fig. 2). Since LBG is a type of polysaccharide, it does not dissolve well in water at 20–30 °C. Therefore, it requires preliminary preparation^23,25–27^. For the preparation, LBG powder was mixed with Sodium hydroxide and distilled water in a 250 mL conical flask and placed in a hotplate, continuously stirring using a magnetic stirrer until a homogeneous opaque liquid was used obtained.

Dodecylamine CH_3_(CH_2_)_11_NH_2_ was considered as the collector. Because long-chain amines are only slightly soluble in water, they were dissolved using hydrochloric acid^29^. It should be noted that HCl (hydrochloric acid) was used to prepare the collector and pH modifier, and NaOH is also used as a pH regulator. All solutions were prepared with specified concentrations using distilled water.

### Micro-flotation (pure minerals)

The micro-flotation tests for the single pure minerals were carried out in an 80 mL Hallimond tube. The fixed speed of the mechanical stirrer and the aeration rate were 650 rpm and 100 mL/min, respectively. In all experiments, 1.0 g of pure minerals and around 75 mL of distilled water were mixed in the tube and were conditioned for 1 min under constant agitation. The solution's pH was set at 10. After that, depressant (for 3 min) and collector solution (for 1 min) were added, respectively. Finally, flotation was carried out for 1 min. The pH level was monitored throughout the conditioning process. The flotation product was dried in an oven at 50 °C and accurately weighed before further characterization. Each condition through micro-flotation tests was repeated five times to ensure the results’ reproducibility.

### Batch flotation (mixed minerals)

The mineral mixture test was carried out in the mass ratio of 75:25 "hematite: quartz". The batch flotation test was conducted using a 1 L Denver D12 laboratory flotation cell. The solid percentage and the agitation rate were set at 30% and 1100 rpm, respectively. The airflow rate was 5 L/min. The collector and depressant concentrations were used at 150 and 300 g/t, respectively. The same conditioning and reagent addition times as the micro-flotation tests were considered, while the flotation time was 2 min. The froth products and tails were dried in an oven at 50 °C and accurately weighed before further characterization. Recovery was calculated based on the dry weight (Eq. 1) and chemical analyses. The experiments were carried out in duplicate, with the average values reported. Moreover, a real ore (Fe total 46.67%, SiO_2_ 13.7%, P 1.7%) was provided, drily milled, and similar experiments were conducted to assess the locust bean gum depression capability.

1 $$R = \frac{(f - t) \times c}{{(c - t) \times t}} \times 100$$

where f, t, and c, are grade of Fe on the feed, tail and concentrate.

### Surface wettability

Contact angle measurements were conducted to characterize the surface wettability of minerals in both the absence and presence of reagents. The Sessile Drop Method (SDM) was used to determine the contact angle using the goniometer DSA25 (provided by Kruss, Germany). The minerals plate surface was pre-conditioned with flotation reagents. Subsequently, using a glass syringe and a needle with diameters of 0.510 and 0.487 mm, a water droplet was gently deposited on the mineral's surface to determine the contact angle. All measurements were taken at room temperature. The Kruss software automatically measures the system's three-phase contact points between the baseline and the fitted bubble shape to provide an accurate contact angle measurement. Because they can fit the bubble boundaries and baseline, the Young–Laplace, ellipse, and circle fitting models were used. . It was generally known that Young–Laplace is the most trustworthy model for measuring tiny angles, whereas ellipse fitting is more accurate for calculating greater contact angle, often exceeding 40°. Hematite and quartz's contact angles were measured both with and without LBG (300 mg/L). Using various concentrations of DDA (0, 5, 30, 50, and 75 mg/L). The work of adhesion and spreading coefficient values were calculated based on Eqs. (2) and (3) using the means of the measured contact angles and their standard deviations for each experimental condition.

2 $$W_{a} = \gamma_{{_{LV} }} (\cos \theta + 1)$$

3 $$S = \gamma_{{_{LV} }} (\cos \theta - 1)$$

where Wa, S, θ, and γ_LV_ represent the work of adhesion (erg/cm2), spreading coefficient (erg/cm2), contact angle (°), and the surface tension of water (mN/m) in the order.

### Surface adsorption

The solution depletion technique was used for adsorption studies. In this procedure, 1.0 g of each pure mineral was added to a 40 mL solution containing a certain concentration of LBG, and the pH was then adjusted to the desired level. For 2 h at room temperature, the flasks were stirred at 220 rpm. The flasks were left standing (still, not shaken) for 1 h to allow the suspended solids was settling naturally. A 25 mL pipette was used to remove the supernatant, which was then centrifuged for 15 min at 6,000 rpm in a lab-scale centrifuge. Thermo gamma metric's Helios Alpha UV–Vis Spectrophotometer was then used to measure the remaining LBG content at a wavelength of 279 nm. The difference between the original and residual concentrations was used to compute the adsorption of LBG on the mineral surfaces (Eq. 4).

4 $${q}_{e}=\frac{({C}_{1}-{C}_{2})\times V}{m}$$

where C_1_ and C_2_ represent the first and final LBG concentrations (mg/L), respectively. q_e_ is the equilibrium adsorption capacity of the adsorbent (mg/g), V is the volume of the solution (L), and m is the weight of the minerals (g). Freundlich and Langmuir adsorption isotherms were applied to understand the LBG adsorption mechanisms on the mineral's surface (Eqs. 5 and 6, respectively). The n and K_F_ factors for the Freundlich adsorption isotherm and the qm and K_L_ factors for the Langmuir adsorption isotherm were determined, respectively.

5 $$log \, q_{e} = \, log \, K_{F} + \, 1/n \, log \, C_{e }$$

6 $$1/q_{e} = 1/q_{m} + \, 1/K_{L} qmC_{e}$$

### Zeta potential measurement

The zeta potential on the mineral's surface was measured using a Zetasizer Nano ZS. Zeta potential measurements were performed at pH values of 2, 4, 7, 9, 10, and 11 in the presence of 300 mg/L LBG and its absence. 1 g of mineral sample was added to a 100 mL pre-conditioned solution. A magnetic stirring was used to condition the suspension. The pH level was monitored and maintained during conditioning. A digital pH-meter electrode was positioned within the solution during conditioning, and the pH level was continuously checked. The suspension was allowed to stand for 5 min for settling the particles. A 3 mL supernatant sample was taken out and used to determine the zeta potential. The findings of all tests, which were all conducted at room temperature, were the mean of three separate measurements.

### FT-IR analysis

The Fourier transform infrared (FTIR) spectrometry was applied to discover the molecular structures and the functional groups on the surface of pure single minerals before and after conditioning with LBG. In order to condition the samples, 1.0 g of each pure sample was added to an aqueous solution containing 300 mg/L of LBG, and the samples were then conditioned for 6 h (pH 10). The particles were filtered and dried for 24 h at room temperature. 1% weight of KBr (potassium bromide) was added to the mineral sample. The spectra of pure minerals (untreated) were also analyzed to make a comparison.

## Results

### Flotation

#### Micro-flotation

**Figure 3:**
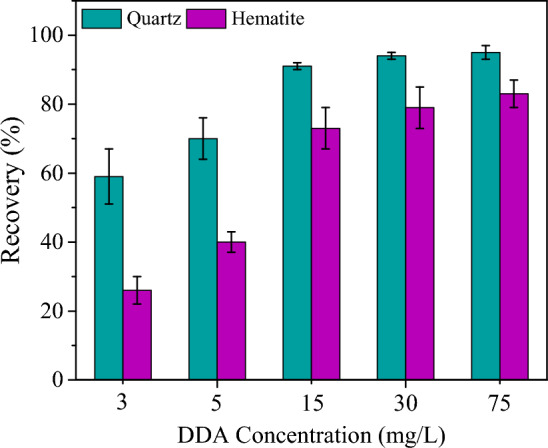
Effect of DDA on the floatability of pure hematite and quartz in the absence of depressant at pH = 10.

**Figure 4:**
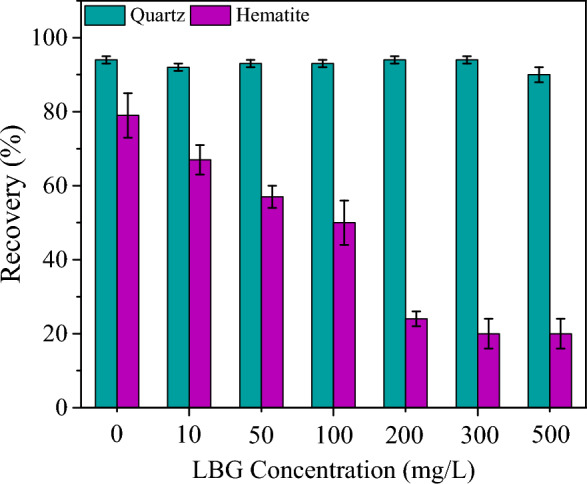
Effect of LBG on the floatability of pure hematite and quartz (in the presence of collector; 30 mg/L of DDA) at pH = 10.

Hematite reverse flotation separation from quartz is commonly known to take place at pH 10^30^. Micro-flotation test results (Fig. 3) at a DDA concentration of 30 mg/L showed that the recovery of quartz and hematite were 94 and 75%, respectively. The recovery of quartz and hematite did not significantly change till the DDA concentration reached 75 mg/L. Micro-flotation outcomes released that without the addition of LBG, quartz, and hematite both would be floated even at the low DDA concentration (Fig. 3). However, by adding and increasing the LBG concentration, the floatability of hematite was significantly dropped (Fig. 4). While the quartz floatability and recovery showed a negligible decrease (Fig. 4). The LBG dosage was set at 300 mg/L since the hematite depression did not markedly improve above it.

#### Batch flotation

**Figure 5:**
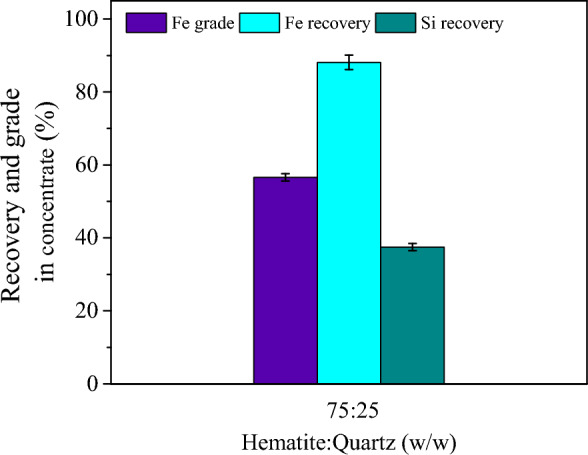
Fe grade, Iron recovery, and Si recovery of achieved iron concentrate from hematite and quartz mixtures flotation (in the presence of 150 g/t DDA, 300 g/t LBG, and pH value of 10).

Batch flotation test with a mass ratio of 75:25 "hematite: quartz" was conducted (Fig. 5). Batch flotation outcomes highlighted that the Fe grade, Fe recovery, and Si recovery in concentrate were 56.6, 88.1, and 37.5%, respectively. These results generally agreed with the previous investigations, indicating a reasonable grade and recovery could be obtained with a single-stage flotation^1,14,16^. These findings showed that LBG could selectively depress hematite and enhance flotation efficiency. Experiments on the real ore samples indicated that Fe recovery through the rougher and cleaner stage would be 85.41%.

### Surface wettability

**Figure 6:**
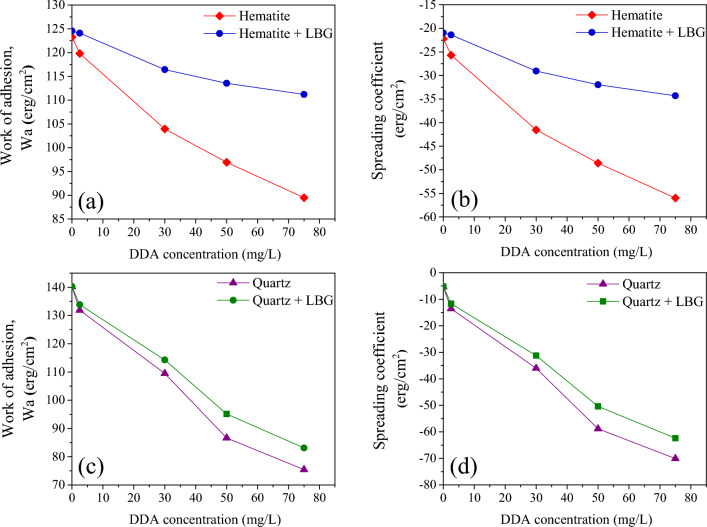
Wettability parameters of pure quartz and hematite in the presence or absence of 300 mg/L LBG at pH = 10 as a function of DDA concentration.

The surface wettability of quartz and hematite was investigated by measuring their work of adhesion based on contact angle in the presence and absence of LBG and as a function of DDA concentration. Results (Fig. 6a–d) showed that increasing collector concentration reduced the work of adhesion for both minerals, indicating that DDA decreased their surface energy. Other studies have also reported similar findings where the Wa values for aqueous solutions decreased as the concentration of cationic collectors increased^31^.

The results of wettability were consistent with the micro-flotation outcomes, which demonstrated that increasing collector concentration improved the floatability of both quartz and hematite minerals (Fig. 3). Lelis et al. (2019, and 2022) showed that the quartz surface energy was getting significantly lower than the hematite by increasing cationic collector concentrations^32,33^. These data illustrated the LBG-treated quartz was more hydrophobic. The spreading coefficient products, which show how one liquid spreads over a solid phase, showed a comparable pattern for both quartz and hematite minerals. A high negative spreading coefficient value is preferred for flotation separation^34^. It was indicated that the hydrophobicity of the quartz was considerably greater than the treated hematite with LBG, which supported the results of the micro-flotation test. The observed phenomena in the study, where the quartz mineral exhibited higher hydrophobicity than the LBG-treated hematite mineral, may be attributed to the DDA ions having a greater electrostatic attraction to the quartz surface than the LBG-treated hematite surface. This electrostatic attraction may have been further enhanced by forming hydrogen bonds between the DDA ions and the silanol groups on the quartz surface^32,35–37^. Surface wettability outcomes (Fig. 6a) also indicated that the LBG-treated hematite had a higher work of adhesion than the untreated hematite samples. This high adhesion could be correlated to a higher affinity of the LBG-treated hematite for water. In other words, even in the presence of DDA, LBG significantly depressed the hematite surface compared to quartz. In the presence of the collector, the spreading coefficient of the LBG-treated hematite further demonstrated that its surface was turned to be fully wet. On the other hand, quartz exhibited a different response (Fig. 6d), with lower levels of its spreading and adhesion work in the presence of LBG than hematite. It was found that the addition of LBG only slightly changed the wettability of quartz in the presence of DDA. These results support the micro-flotation findings, which showed that quartz showed high floatability even when LBG was present (Fig. 4).

### Surface adsorption

**Figure 7:**
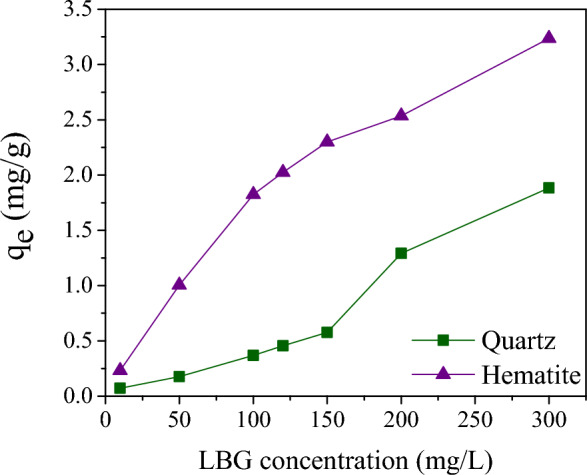
Adsorption amount of LBG on the hematite and quartz as a function of LBG concentration at pH = 10 (particle size: + 38–75 μm).

Based on the assessment of LBG adsorption on the surfaces of quartz and hematite, it was found that increasing the LBG concentration resulted in an increase in the amount of adsorbed LBG on both mineral surfaces (as shown in Fig. 7). However, the amount of LBG adsorbed on the hematite surface was much higher compared to that on the quartz surface, even across a wide range of LBG concentrations. Specifically, when the LBG concentration reached 150 mg/L, the adsorption quantity of LBG on the hematite surface was 2.3 mg/g, while on the quartz surface, it was only 0.57 mg/g at the same concentration. This indicates that LBG has a stronger adsorption interaction with the hematite surface than with quartz.

Additionally, the adsorption equilibrium data were analyzed using the Freundlich and Langmuir isotherm equations, and the results (as shown in Table 3) indicated that the Langmuir isotherm model was a more appropriate fit for declaring LBG adsorption on the mineral surfaces due to its higher correlation coefficients. Furthermore, the q_m_ values of hematite and quartz were found to be 3.90 and 0.86, respectively, suggesting that the interaction of LBG with hematite is significantly stronger than that with quartz.

**Table 3:** Freundlich and Langmuir constants of LBG adsorption on hematite and quartz surfaces (particle size: + 38–75 μm).

Minerals	Langmuir parameters			Freundlich parameters		
	q_m_	K_L_	R^2^	n	K_F_	R^2^
Hematite	3.90	0.015	0.99	1.48	0.10	0.97
Quartz	0.86	0.01	0.95	1.07	0.0073	0.90

### Zeta potential measurement

**Figure 8:**
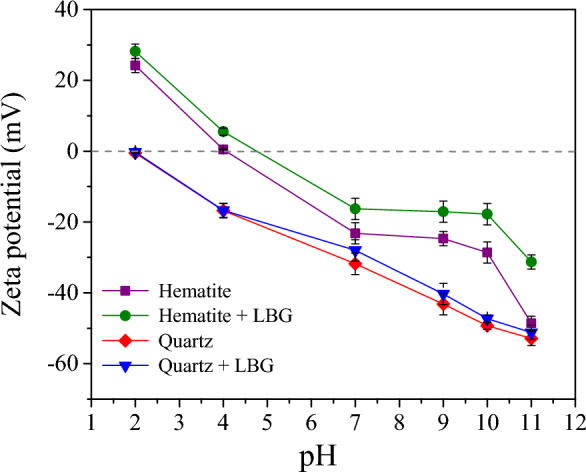
Zeta potentials of hematite and quartz at different pH values in the presence of 300 mg/L LBG.

The zeta potential measurements (Fig. 8) indicated that bare quartz and hematite's IEP (isoelectric point) occurred at pH 2 and about 4.2, respectively. Similar values were reported in various investigations^16,38–41^. The zeta potentials on the surface of both hematite and quartz virtually remained more negative as the pH values increased and were negative over the flotation pH range. However, when LBG was added (300 mg/L), the zeta potentials of both treated minerals were increased compared to their untreated minerals. The variation on the hematite surface in the presence of LBG was higher than in quartz, indicating that LBG was adsorbed more on the hematite surface. These results are compatible with the outcomes of wettability and adsorption analyses. However, the magnitude of surface charge for both minerals remained unaffected. This phenomenon could be due to the non-ionic polymeric properties of LBG, a polysaccharide with many hydroxyl groups with non-ionic polymeric characteristics^42^. The observed variations were caused by the movement of the sliding plates of double electric layers at the mineral interfaces affected by LBG adsorbed onto the surfaces of both minerals^24,43^.

### FT-IR analysis

**Figure 9:**
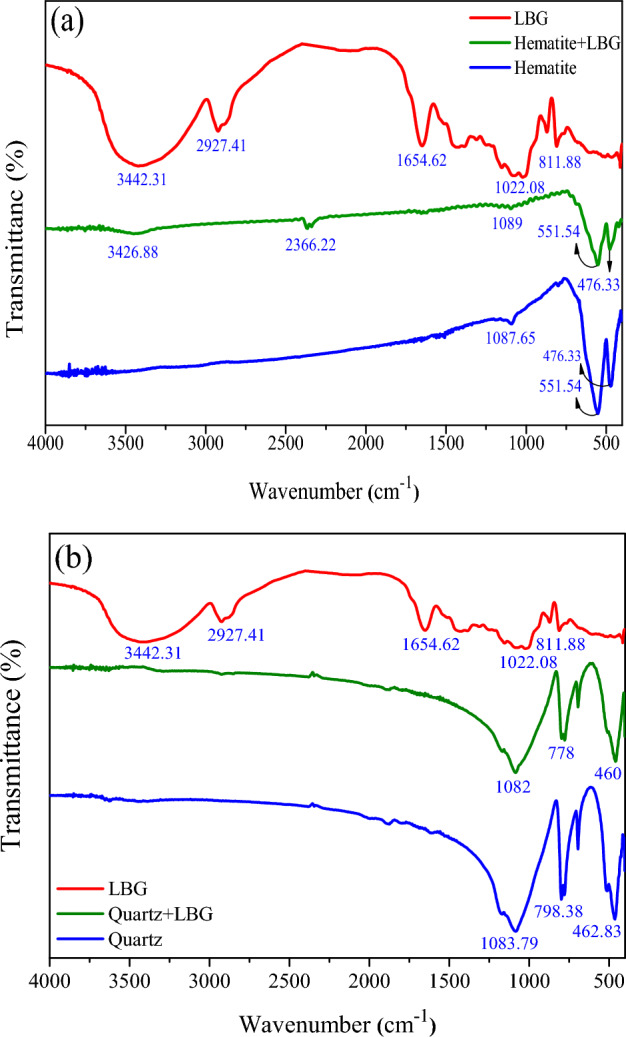
FT-IR spectra of (**a**) Hematite and (**b**) Quartz treated with 300 mg/L of LBG.

According to the FT-IR analyses (as shown in Fig. 9), the spectrum of LBG displayed a broad band at 3425.41 cm^−1^, which is associated with the stretching vibration of –OH groups. The C–H stretching vibration of alkyl –CH and –CH_2_ groups were observed at 2927.41 cm^−1^, and the C–O–H stretching vibration was present at 1022.08 cm^−1^ ^44,45^. The chemical structures of LBG contain oxygen-containing functional groups, such as carboxyl and hydroxyl, which cause LBG to interact with the surfaces of metallic minerals. As seen in (Fig. 9a), the characteristic bands of hematite appeared at 476.33 cm^−1^, 551.54 cm^−1^, and 1087.45 cm^−1^, which were related to the Fe–O vibration (Metal-O) and –OH stretching vibration^46^. When LBG-treated hematite surface showed (Fig. 9a), new peaks appeared at 3426.88 and 2366.22 cm^−1^, related to the stretching vibration of hydroxyl groups –OH groups and the stretching vibrations of the –CH_2_ from the LBG spectrum, respectively. These results suggest that LBG molecules were effectively adsorbed on the hematite surface. The presence of many hydroxyl groups in LBG's structure may have facilitated hydrogen bonding, making adsorption between LGB and the hematite surface possible. On the other hand, characteristic bands for quartz particles were observed at 1083.79 cm^−1^, 798.38 cm^−1^, and 462.83 cm^−1^ (silanol groups and –OH bands) (Fig. 9b). When quartz was treated with LBG, no new characteristic peaks appeared in the quartz + LBG spectrum (Fig. 9b). These spectra and zeta potential measurement demonstrated that LBG interacts weakly with the quartz surface. Thus, LBG can be used as a selective depressant of hematite in flotation separation from quartz.

## Discussion

According to the results, LBG has the potential to act as an effective hematite depressant in reverse cationic flotation. Results obtained from micro-flotation tests indicated that LBG could significantly decrease the floatability of hematite while having a negligible impact on the recovery of quartz. Surface characterization analyses showed that the hydrophobicity of quartz was considerably higher than that of hematite when treated with LBG, which corroborated the results of the micro-flotation experiments (Fig. 3).

Based on the Wettability data (Fig. 6), the surface energy of quartz is much lower than hematite. Hematite is exposed to Fe^3+^ and O^2−^ on its surface, while quartz has Si^4+^ and O^2−^. A high proportion of Si^4+^ cations and a low proportion of metallic cations on the quartz surface increased the adsorption of amine species and, consequently, improved floatability^47^. In contrast, metal sites on mineral surfaces play a significant role in the adsorption of depressants. The result showed that the quantity of LBG adsorbed on the surface of hematite was significantly greater than that on the quartz surface. Based on the zeta potential measurement (Fig. 8), LBG has fewer interactions with the quartz surface. When LBG add to the process, the hematite zeta potential becomes less positive, increasing the potential difference between hematite and quartz and improving separation. However, due to the unfavorable basicity and the constant IEP of quartz at a pH of 2, LBG generally does not interact with quartz (Fig. 8). Conversely, hematite interacts more strongly with high polysaccharide adsorption densities because it has an IEP of 4.2 pH, according to zeta potential tests. Thus, the LBG's adsorption on the hematite surface is attributed to the interaction between the metallic ions on the hematite surface and the anionic functional groups of LBG.

These findings are supported by FTIR analysis (Fig. 9). The chemical structure of LBG includes oxygen-containing functional groups, carboxyl, and hydroxyl, which were detected in the FTIR spectra. In an alkaline environment, numerous free carboxyl groups were present in the LBG solution, which has a potent complexing effect with multivalent metal ions^46^. Thus, LBG had a higher affinity for the hematite surface than quartz. The adsorption between LBG and the hematite surface was attributed to hydrogen bonding, specifically involving the Fe–O vibration (Metal-O) and –OH stretching vibration. The interaction between polysaccharides and hematite has been characterized as an acid/base interaction, where the polysaccharide acts as an acid, and the hematite surface serves as a base^48,49^. Adsorbing LBG selectively onto the hematite surface would hinder DDA adsorption; thereby, hematite would be selectively depressed. However, since LBG has weak adsorption to quartz, a large amount of DDA was adsorbed on quartz, which aided in achieving a high quartz recovery.

## Conclusions

In this study, the depression effect of locust bean gum (LBG), as a novel and environmentally friendly depressant, for the selective separation of hematite and quartz through reverse cationic flotation was investigated through various flotation conditions. Micro-flotation results illustrated that LBG could significantly reduce the hematite floatability even at a low concentration (30 mg/L) with an insignificant effect on the quartz recovery. The batch flotation experiment revealed that LBG has a high selectivity for hematite depression, with Fe grades and Fe recovery in concentrates of 56.6 and 88.1, respectively. Various surface analyses showed that the LBG adsorption on hematite and quartz differed significantly. The wettability analysis indicated that by increasing the collector concentrations, the surface energy of quartz was significantly lower than hematite; thus, the quartz hydrophobicity was significantly higher than the LBG-treated hematite. Moreover, the difference in adhesion between LBG-treated hematite and untreated samples was very substantial. Adding LBG only slightly changed the wettability of quartz in the presence of DDA. Surface adsorption analysis depicted that LBG interacted with the hematite surface more strongly than quartz, while at the concentration of 300 mg/L LBG, the adsorption quantities of hematite and quartz were 3.3 and 1.7 (mg/g), respectively. The FT-IR outcomes revealed that LBG molecules were adsorbed via hydrogen bonding on the hematite surface and interacted weakly with the quartz surface.

## Data Availability

The datasets used and/or analysed during the current study available from the corresponding author on reasonable request.

## References

[1] Tohry A, Dehghani A (2016). Effect of sodium silicate on the reverse anionic flotation of a siliceous–phosphorus iron ore. Sep. Purif. Technol..

[2] Rocha L, Cançado RZL, Peres AEC (2010). Iron ore slimes flotation. Miner. Eng..

[3] Quast K (2017). Literature review on the use of natural products in the flotation of iron oxide ores. Miner. Eng..

[4] Arantes RS, Lima RMF (2013). Influence of sodium silicate modulus on iron ore flotation with sodium oleate. Int. J. Miner. Process..

[5] Araujo AC, Viana PRM, Peres AEC (2005). Reagents in iron ores flotation. Miner. Eng..

[6] Ma X, Marques M, Gontijo C (2011). Comparative studies of reverse cationic/anionic flotation of Vale iron ore. Int. J. Miner. Process..

[7] Yang B (2021). Selective collection performance of an efficient quartz collector and its response to flotation separation of malachite from quartz. Miner. Eng..

[8] Wang L (2021). Selective separation of hematite from quartz with sodium oleate collector and calcium lignosulphonate depressant. J. Mol. Liq..

[9] Zhang M, Xu Z, Wang L (2022). Ultrasonic treatment improves the performance of starch as depressant for hematite flotation. Ultrason. Sonochem..

[10] Wang D, Liu Q (2021). Influence of aggregation/dispersion state of hydrophilic particles on their entrainment in fine mineral particle flotation. Miner. Eng..

[11] Qi GW, Klauber C, Warren LJ (1993). Mechanism of action of sodium silicate in the flotation of apatite from hematite. Int. J. Miner. Process..

[12] Shrimali K, Miller JD (2016). Polysaccharide depressants for the reverse flotation of iron ore. Trans. Indian Inst. Met..

[13] Poperechnikova OY, Filippov LO, Shumskaya EN, Filippova IV (2017). Intensification of the reverse cationic flotation of hematite ores with optimization of process and hydrodynamic parameters of flotation cell. J. Phys. Conf. Ser..

[14] Tohry A, Dehghan R, Zarei M, Chelgani SC (2021). Mechanism of humic acid adsorption as a flotation separation depressant on the complex silicates and hematite. Miner. Eng..

[15] dos Santos ID, Oliveira JF (2007). Utilization of humic acid as a depressant for hematite in the reverse flotation of iron ore. Miner. Eng..

[16] Tohry A, Dehghan R, de Salles Leal Filho L, Chehreh Chelgani S (2021). Tannin: An eco-friendly depressant for the green flotation separation of hematite from quartz. Miner. Eng..

[17] Mineralurgii FP (2002). Dextrins As Selective Flotation..

[18] Veloso CH, Filippov LO, Filippova IV, Ouvrard S, Araujo AC (2018). Investigation of the interaction mechanism of depressants in the reverse cationic flotation of complex iron ores. Miner. Eng..

[19] Li K, Zhang H, Peng T, Liu C, Yang S (2022). Influences of starch depressant with the various molecular structure on the interactions between hematite particles and flotation bubbles. Colloids Surfaces A Physicochem. Eng. Asp..

[20] Yang S, Li C, Wang L (2017). Dissolution of starch and its role in the flotation separation of quartz from hematite. Powder Technol..

[21] Zhang J, Yang C, Niu F, Gao S (2022). Molecular dynamics study on selective flotation of hematite with sodium oleate collector and starch-acrylamide flocculant. Appl. Surf. Sci..

[22] Prajapati VD (2013). Galactomannan: A versatile biodegradable seed polysaccharide. Int. J. Biol. Macromol..

[23] Shen Z (2021). Selective depression mechanism of locust bean gum in the flotation separation of chalcopyrite from pyrite in a low-alkalinity media. Miner. Eng..

[24] Feng B (2018). Use of locust bean gum in flotation separation of chalcopyrite and talc. Miner. Eng..

[25] Feng B (2020). Effect of surface oxidation on the depression of sphalerite by locust bean gum. Miner. Eng..

[26] Miao Y, Wen S, Shen Z, Feng Q, Zhang Q (2022). Flotation separation of chalcopyrite from galena using locust bean gum as a selective and eco-friendly depressant. Sep. Purif. Technol..

[27] Feng B (2019). Flotation separation behavior of chalcopyrite and sphalerite in the presence of locust bean gum. Miner. Eng..

[28] Feng B (2018). Synergistic effect of acidified water glass and locust bean gum in the flotation of a refractory copper sulfide ore. J. Clean. Prod..

[29] Partridge AC, Smith GW (1971). Flotation and adsorption characteristics of the hematite-dodecylamine-starch system. Can. Metall. Q..

[30] Houot R (1983). Beneficiation of iron ore by flotation—Review of industrial and potential applications. Int. J. Miner. Process..

[31] Zhang L (2010). Wettability of a Quartz surface in the presence of four cationic surfactants. Langmuir.

[32] Lelis DF, da Cruz DG, Fernandes Lima RM (2019). Effects of calcium and chloride ions in iron ore reverse cationic flotation: fundamental studies. Miner. Process. Extr. Metall. Rev..

[33] Lelis DF, Fernandes Lima RM, Rocha GM, Leão VA (2022). Effect of magnesium species on cationic flotation of quartz from hematite. Miner. Process. Extr. Metall. Rev..

[34] Leja J (1982). Surface Chemistry of Froth Flotation.

[35] Smith RW, Scott JL (2007). Mechanisms of dodecylamine flotation of quartz mechanisms of dodecylamine flotation of quartz. Miner. Process. Extr. Metall. Rev..

[36] Vidyadhar A, Rao KH, Chernyshova IV, Pradip & Forssberg, K. S. E.  (2002). Mechanisms of amine-quartz interaction in the absence and presence of alcohols studied by spectroscopic methods. J. Colloid Interface Sci..

[37] Churaev NV, Sergeeva IP, Sobolev VD, Jacobasch H (2000). Modification of quartz surfaces using cationic surfactant solutions..

[38] Rao, S. R. *Surface Chemistry of Froth Flotation.* Springer, New York (2004). doi:10.1007/978-1-4757-4302-9.

[39] Carlson JJ, Kawatra SK (2013). Factors affecting zeta potential of iron oxides. Miner. Process. Extr. Metall. Rev..

[40] Rohem Peçanha, E., da Fonseca de Albuquerque, M. D., Antoun Simão, R., de Salles Leal Filho, L. & de MelloMonte, M. B. Interaction forces between colloidal starch and quartz and hematite particles in mineral flotation. *Colloids Surfaces A Physicochem. Eng. Asp.* **562**, 79–85 (2019).

[41] Fan G, Wang L, Cao Y, Li C (2020). Collecting agent–mineral interactions in the reverse flotation of iron ore: A brief review. Minerals.

[42] Tang M, Lei Y, Wang Y, Li D, Wang L (2021). Rheological and structural properties of sodium caseinate as influenced by locust bean gum and κ-carrageenan. Food Hydrocoll..

[43] Huang P, Wang L, Liu Q (2014). Depressant function of high molecular weight polyacrylamide in the xanthate flotation of chalcopyrite and galena. Int. J. Miner. Process..

[44] Kaity S, Isaac J, Ghosh A (2013). Interpenetrating polymer network of locust bean gum-poly (vinyl alcohol) for controlled release drug delivery. Carbohydr. Polym..

[45] Jana S, Sen KK (2017). Chitosan — Locust bean gum interpenetrating polymeric network nanocomposites for delivery of aceclofenac. Int. J. Biol. Macromol..

[46] Pavlovic S, Brandao PR (2003). Adsorption of starch, amylose, amylopectin and glucose monomer and their effect on the flotation of hematite and quartz. Miner. Eng..

[47] Severov VV, Filippov LO, Filippova IV (2016). Relationship between cation distribution with electrochemical and flotation properties of calcic amphiboles. Int. J. Miner. Process..

[48] Liu Q, Zhang Y, Laskowski JS (2000). The adsorption of polysaccharides onto mineral surfaces: an acid/base interaction. Int. J. Miner. Process..

[49] Laskowski JS, Liu Q, O’Connor CT (2007). Current understanding of the mechanism of polysaccharide adsorption at the mineral/aqueous solution interface. Int. J. Miner. Process..

